# A novel perspective for burn-induced myopathy: Membrane repair defect

**DOI:** 10.1038/srep31409

**Published:** 2016-08-22

**Authors:** Chao Wang, Hongyu Wang, Dan Wu, Jianhong Hu, Wei Wu, Yong Zhang, Xi Peng

**Affiliations:** 1 Institute of Burn Research, State Key Laboratory of Trauma, Burns and Combined Injury, Southwest Hospital, Third Military Medical University, Chongqing 400038, China

## Abstract

Myopathy is a common complication of severe burn patients. One potential cause of this myopathy could be failure of the plasma membrane to undergo repair following injuries generated from toxin or exercise. The aim of this study is to assess systemic effect on muscle membrane repair deficiency in burn injury. Skeletal muscle fibers isolated from burn-injured mice were damaged with a UV laser and dye influx imaged confocally to evaluate membrane repair capacity. Membrane repair failure was also tested in burn-injured mice subjected to myotoxin or treadmill exercise. We further used C2C12 myotubules and animal models to investigate the role of MG53 in development of burn-induced membrane repair defect. We demonstrated that skeletal muscle myofibers in burn-injured mice showed significantly more dye uptake after laser damage than controls, indicating a membrane repair deficiency. Myotoxin or treadmill exercise also resulted in a higher-grade repair defect in burn-injured mice. Furthermore, we observed that burn injury induced a significant decrease in MG53 levels and its dimerization in skeletal muscles. Our findings highlight a new mechanism that implicates membrane repair failure as an underlying cause of burn-induced myopathy. And, the disorders in MG53 expression and MG53 dimerization are involved in this cellular pathology.

Severe burns continue to be a major medical problem globally, with significant mortality and disability resulting from a devastating impairment perturbing nearly every organ system^1^,^2^. It has been established that burn injury increases the possibility of development of systemic myopathy characterized by muscle wasting, muscle weakness, and altered bioenergetics. These acquired myopathic derangements result in difficulty in weaning off the ventilator, prolonged mechanical ventilation, pulmonary infection, exercise limitation, metabolic disorders, and poor quality of life^3^,^4^,^5^. The mechanisms leading to these muscular changes are linked to the high-grade of inflammatory factors in plasma, and activation of proteolytic pathways in skeletal muscles^6^,^7^. However, abundant evidence indicates that the mechanisms responsible for myopathy in burn patients could be complex and multifactorial^8^,^9^. To our knowledge, skeletal muscle membrane integrity is commonly recognized as a main factor in the pathogenesis of myopathy^10^. Not unexpectedly, the myocyte membranes have been shown to be injured by thermal force at the site of burn. However, the changes of integrity in sarcolemmal membrane distant from the site of burn injury remain unclear.

Plasma membrane injuries are naturally appearing in mechanically active organs, such as cardiac and skeletal muscle. Skeletal muscle contractions, especially high-force eccentric exercise, disrupt the myocyte membranes of the contracting myofibers. However, these disruptions are transient and rarely fatal injuries because they can trigger in the injured myofibers a rapid membrane repair response which is initiated by the influx of extracellular molecules^11^. Briefly, the influx of extracellular molecules initiates fusion of internal plasma membrane, thus forming a “patch vesicle”, which is then accurately transported to the injured site to reform myocyte membrane^12^. During the life cycle of skeletal muscle, membrane repair response is a fundamental process in maintaining membrane continuity, as demonstrated by recent studies that link defective membrane repair to the progression of muscle membrane injury^10^.

Reportedly, many proteins are essential for the diverse fusion events of membrane repair response. Thus, almost all muscle diseases induced by membrane repair failure are demonstrated to be associated with these proteins^11^. MG53 belongs to the Tripartite motif (TRIM) family of proteins and is one of the several membrane proteins that participate in the membrane repair response^13^,^14^. These skeletal muscles lacking MG53 display increased membrane permeability, which ultimately result in serious myopathy^15^,^16^. It has been reported that MG53 is transcriptionally activated by IRS-1/PI3K/Akt signal pathway in skeletal muscle fibers^17^. However, this signal pathway is always inhibited in skeletal muscles for prolonged periods of time after burn injury^18^. Consequently, there is a possibility that skeletal muscle membrane in burn injury would be fragile because of MG53 deficiency.

Here, we hypothesized that sustained musculoskeletal abnormalities that occur in burn patients are caused by on-going membrane injury. As a test of this hypothesis, we extracted skeletal muscles distant from the site of burn injury, and assessed systemic effect on musculoskeletal membrane integrity and musculoskeletal membrane repair deficiency in burn injury using various methodologies. We further used an *in vivo* and *in vitro* model of burn injury to investigate the underlying molecular mechanisms associated with defective membrane repair in burn-injured mice.

## Results

### *In vivo* analysis of muscle membrane integrity after burn in mice

**Figure 1:**
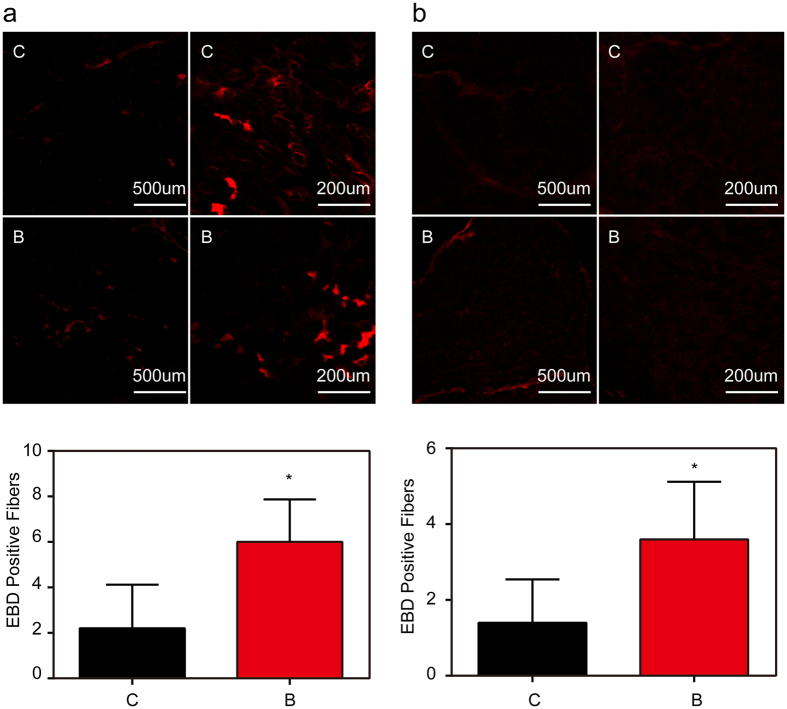
*In vivo* analysis of sarcolemmal membrane integrity after burn injury in mice. (**a**) EBD was intramuscularly injected into control and burn injured (Post Burn Day 14) mice. Membrane-injured myofibers in gastrocnemius muscles from sham injury (C) and burn injury (B) in mice are labeled. The number of EBD-positive myofibers counted from these micrographs. *P < 0.05; vs. Control; n = 5. (Scale bar, 500 μm; 200 μm). (**b**) EBD was intraperitoneally injected into control and burn injured (Post Burn Day14) mice. Membrane-injured myofibers in gastrocnemius muscles from sham injury (C) and burn injury (B) in mice are labeled. The number of EBD-positive myofibers counted from these micrographs. *P < 0.05; vs. Control; n = 5 (Scale bar, 500 μm; 200 μm).

To determine whether myocytes membrane integrity is affected in distant muscles as a result of burn injury, we monitored gastrocnemius muscle myofibers permeability by exploiting Evans Blue dye (EBD). Mice were intraperitoneally or intramuscularly injected with this dye to identify injured and “leaky” myofibers membrane. Gastrocnemius muscle sections from burn-injured mice displayed significantly more areas of EBD staining when compared with sham-injured mice (Fig. 1a,b). Quantitative analysis of total EBD within extracted gastrocnemius muscle bundles provided direct validation to the above results and displayed a significant increase in dye after burn injury (Table 1). Taken together, these results reveal the presence of musculoskeletal membrane damage in a distant muscle as a result of burn injury.

### *In vivo*/*ex-vivo* analysis of muscle membrane repair after burn in mice

**Figure 2:**
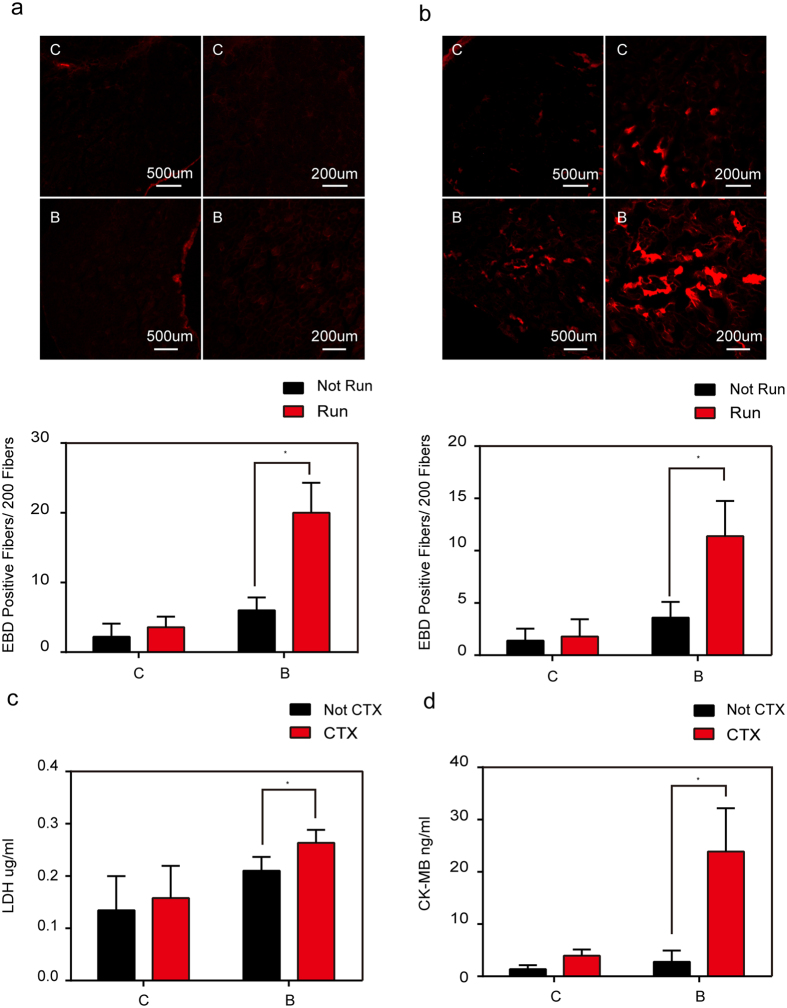
*In vivo* analysis of sarcolemmal membrane repair capacity after burn injury in mice. (**a**) EBD was intramuscularly injected into the mice subjected to treadmill exercise. Myofibers that failed to repair membrane in gastrocnemius muscles from sham injury (C) and burn injury (B) in mice are labeled. The number of EBD-positive myofibers counted from these micrographs. *P < 0.05; n = 5 (Scale bar, 500 μm; 200 μm). (**b**) EBD was intraperitoneally injected into mice subjected to treadmill exercise. Myofibers that failed to repair membrane in gastrocnemius muscles from sham injury (C) and burn injury (B) in mice are labeled. The number of EBD-positive myofibers counted from these micrographs. *P < 0.05; n = 5 (Scale bar, 500 μm; 200 μm). (**d**) CTX was intramuscularly injected into mice. Mice serum was collected for ELISA tests for the levels of LDH. *P < 0.05; n = 3. (**e**) CTX was intramuscularly injected into mice. Mice serum was collected for ELISA tests for the levels of CK-MB. *P < 0.05; n = 3.

To test if myocytes membrane injury is exacerbated in burn-injured mice during eccentric contraction, our study measured gastrocnemius muscle myofibers permeability by EBD in mice subjected to treadmill exercise^11^,^19^,^20^. Compared with non-exercised controls, sham-injured mice demonstrated no significant changes in EBD staining after downhill running (Fig. 2a,b). In contrast, burn-injured mice showed significantly more EBD staining after downhill running, when compared with non-exercised controls (Fig. 2a,b). These findings were supported by quantitative analysis of total EBD within extracted gastrocnemius muscle bundles (Table 1). We next injected myotoxin from Naja mossambica (CTX)^19^, a toxin that injures myofibers membrane, into the gastrocnemius muscles. At 12 hours post-injection, the serum LDH and CK-MB levels in burn-injured mice significantly increased and provided an effective marker of the disruptions of myocytes membrane (Fig. 2c,d).

**Figure 3:**
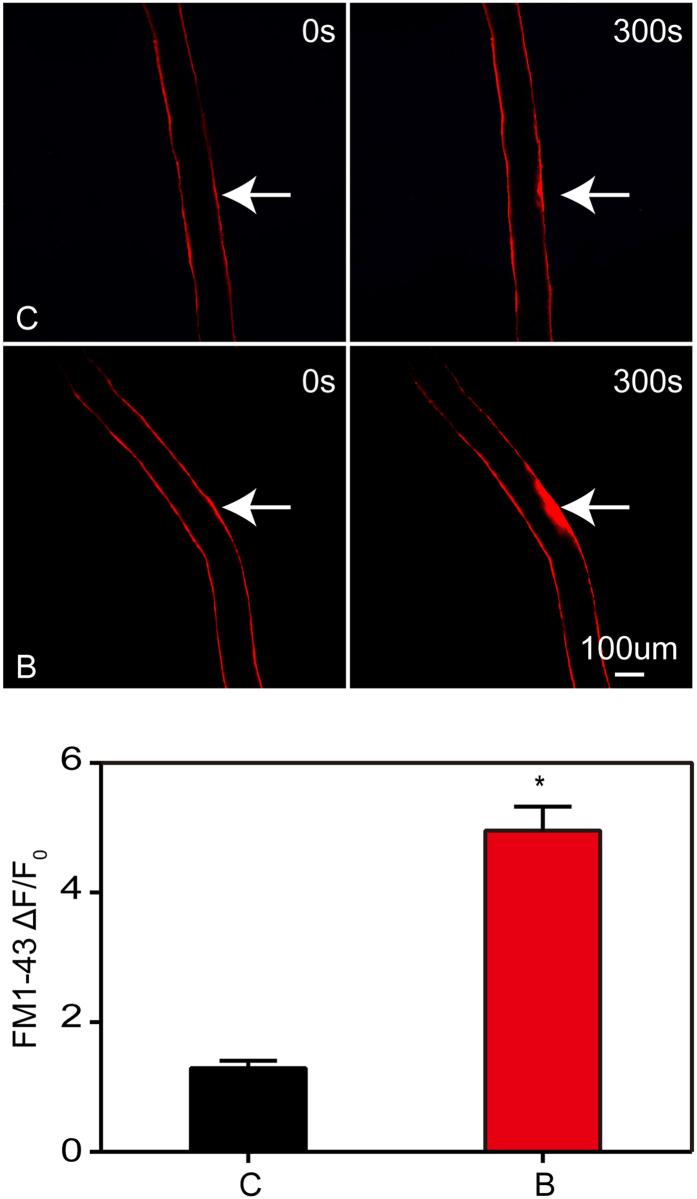
*In ex-vivo* analysis of membrane repair capacity after burn injury in mice. Healthy FDB fibers isolated from sham injury (C) mice exclude FM1-43 dye after laser induced injury, whereas fibers from burn injury (B) mice results in sustained filling of the entire fiber with dye, indicating a membrane repair defect. Arrow indicates damaged site at 300 s after laser injury. An area of about 200 μm adjacent to the injury site was utilized for measuring FM1-43 dye influx fluorescent intensity. Data is presented as ΔF/F_0_. *P < 0.05 vs. Control; n = 3 (Scale bar, 100 μm).

To directly evaluate musculoskeletal membrane repair efficiency of burn-injured mice, we subjected flexor digitorum brevis (FDB) myofibers isolated from burn-injured and sham mice to laser-induced injury and monitored membrane permeability via FM1-43 fluorescent dye entry^20^. FM1-43 strongly and selectively stains the plasma membrane of intact myocytes, however, this pattern can be disrupted with laser damage, which causes membrane disruption and diffusion of the dye to the cytosol. We demonstrated that normal myofibers from sham-injury mice could rapidly and effectively reform injured membrane, as they only showed minimal FM1-43 dye influx after ultraviolet laser damage (Fig. 3). In contrast, we observed more FM1-43 fluorescent dye entry in myofibers from burn-injured mice after laser-induced damage (Fig. 3).

Thus, both *in vivo* and *ex-vivo* model systems strongly support our hypothesis that muscle membrane repair fails in a distant muscle as a result of burn injury.

### Effects of burn injury on MG53 expression and dimerization

**Figure 4:**
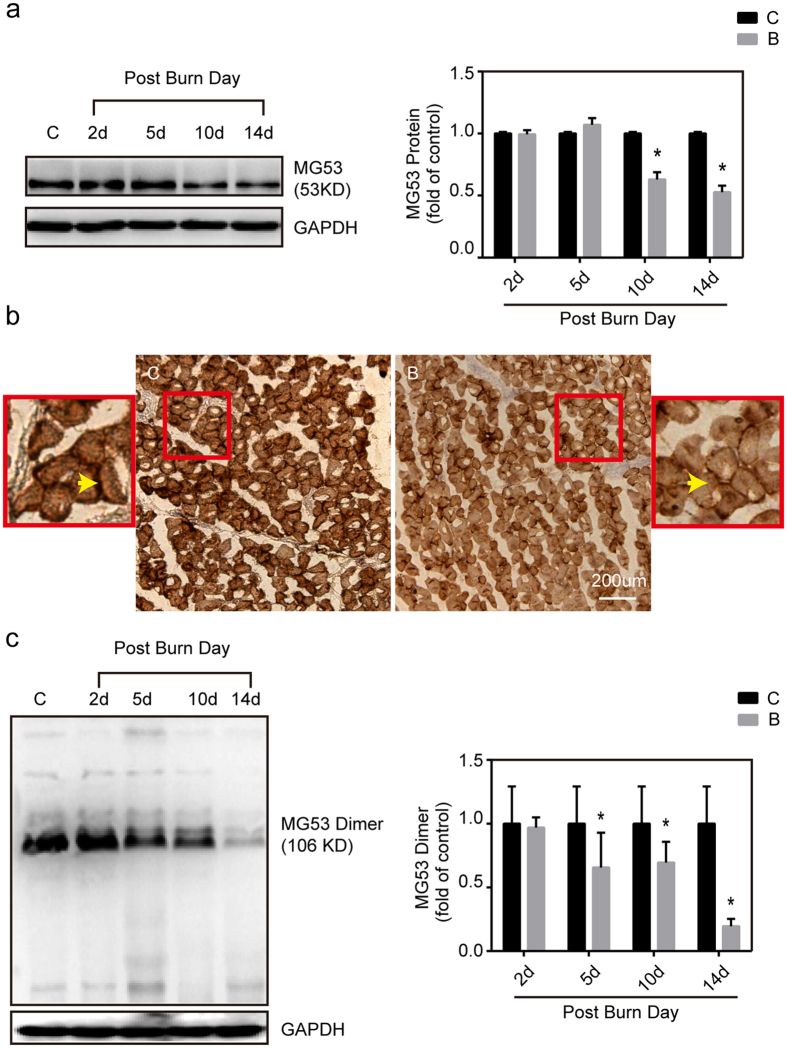
*In vivo* analysis of MG53 expression and dimerization after burn in mice. (**a**) Gastrocnemius muscles were collected at 2, 5, 10 and 14 d after burn injury. Western blot analysis of MG53 protein levels with MG53 antibodies. Data were normalized to the mean control value as determined by the sham-injured group. *P < 0.05 vs. Control; n = 3. (**b**) Immunohistochemical analysis of MG53 protein in the gastrocnemius muscle of sham control (C) and burn injured (B) (Post Burn Day 14) mice (Scale bar, 200 μm). (**c**) Gastrocnemius muscles were collected at 2, 5, 10 and 14 d after burn injury. Western blot analysis of MG53 dimer levels with MG53 antibodies in the absence of dithiothreitol (DTT). Data were then normalized to the mean control value as determined by the sham-injured group. *P < 0.05 vs. Control; n = 3.

To investigate whether there was a causal relationship between MG53 and burn-induced sarcolemmal membrane damage, we first assessed endogenous MG53 protein levels in gastrocnemius muscle by western blot and immunohistochemistry analysis. Western blot analysis illustrated the statistically significant decline in MG53 expression in gastrocnemius muscle at 10 and 14 d after burn injury (Fig. 4a). Immunohistochemistry analysis validated that the MG53 protein is mainly localized to the myocytes membrane and this staining pattern is significantly decreased after burn injury when compared with sham controls (Fig. 4b). Furthermore, Western blot analysis (in the absence of DTT) showed that burn injury induced a significant decline in MG53 dimer in muscles at 5, 10 and 14 d after burn injury, compared with sham injury control (Fig. 4c).

### Protein disulfide isomerase (PDI) inactivation disrupts MG53 dimerization

**Figure 5:**
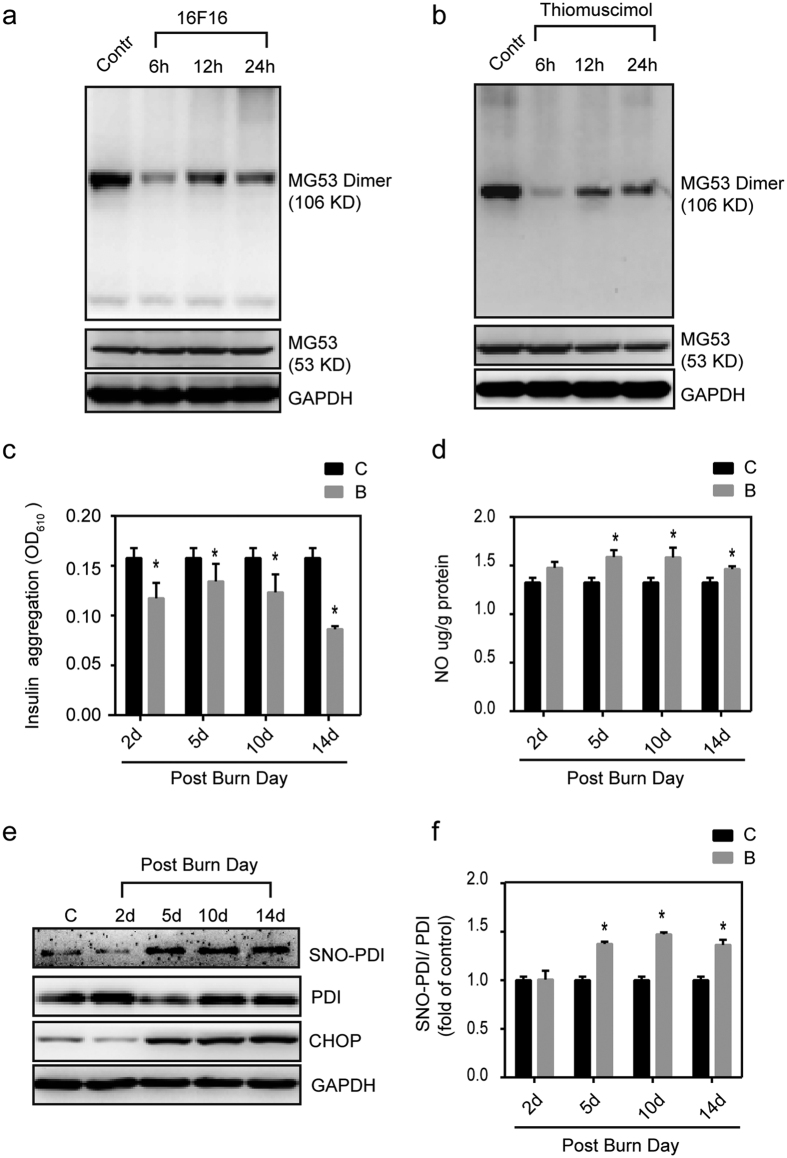
The roles of PDI in MG53 dimerization. (**a**) C2C12 myotubules were cultured for 48 h, and treated with 16F16. After 6, 12 and 24 h of treatment, MG53 dimer levels were assessed by western blot in drug-treated and control cells. (**b**) C2C12 myotubes were cultured for 48 h, and treated with thiomuscimol. After 6, 12 and 24 h of treatment, MG53 dimer levels were assessed by western blot in drug-treated and control cells. (**c**) Gastrocnemius muscle samples were collected at 2, 5, 10 and 14 d after burn injury. PDI activity was measured using an insulin aggregation assay. *P < 0.05 vs. Control; n = 3. (**d**) Gastrocnemius muscle samples were collected at 2, 5, 10 and 14 d after burn injury. The NO levels were measured using an nitric oxide assay kit. *P < 0.05 vs. Control; n = 3. (**e**) Gastrocnemius muscle samples were collected at 2, 5, 10 and 14 d after burn injury. Western blot analysis of PDI, SNO-PDI and CHOP levels with theirs antibodies. SNO-PDI/PDI were normalized to the mean control value as determined by the sham-injured group. *P < 0.05 vs. Control; n = 3.

PDI can catalyze the formation and breakage of disulfide bonds between cysteine residues within many proteins. Lacking functional PDI can increase cellular protein misfolding and thus result in a serious perturbation in dimerization of these proteins^21^. To determine whether the process in MG53 dimerization is affected by PDI, we detected MG53 dimer in C2C12 myotubules after inhibiting PDI activity with 16F16 and thiomuscimol^22^. As illustrated in Fig. 5a,b, inhibition of PDI resulted in a significant decline in MG53 dimer levels in C2C12 myotubules, while showed no significant changes in MG53 protein levels. Furthermore, PDI activity was demonstrated to be reduced in gastrocnemius muscle at 5, 10 and 14 d after burn injury (Fig. 5c), indicating that PDI inactivation could be a possible cause of MG53 dysfunction in burn injury.

Reportedly, NO mediated S-nitrosylation of PDI (SNO-PDI) can inhibit PDI activity and the beneficial effects of PDI^23^. In this study, the NO levels were significantly increased in gastrocnemius muscle after burn injury (Fig. 5d). We further demonstrated that the SNO-PDI levels were significantly increased in gastrocnemius muscle after burn injury (Fig. 5e).

Moreover, it has been established that PDI can be oxidized and lose its activity in presence of endoplasmic reticulum stress (ERS)^24^ In this study, we illustrated that CCAAT/Enhancer-Binding Protein Homologous Protein (CHOP), a major ERS marker, was significantly increased in gastrocnemius muscle at 5, 10 and 14 d after burn injury (Fig. 5e).

### MG53-deficiency induced by insulin insensitivity

**Figure 6:**
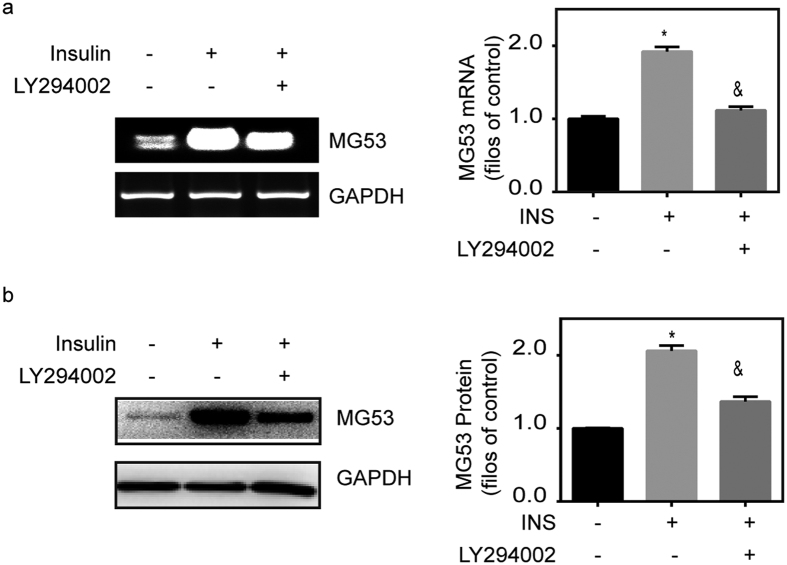
Insulin regulates MG53 expression via PI3K. (**a**) C2C12 myotubes were cultured for 48 h, and treated with insulin (INS, 200 ng/ml) in the absence or presence of LY294002 (10 mM). After 6 h of treatment, MG53 transcript levels were assessed by RT-PCR in drug-treated and control cells. *P < 0.05 vs. Control. &P < 0.05 vs. Insulin; n = 3. (**b**) C2C12 myotubes were cultured for 48 h, and treated with insulin (INS, 200 ng/ml) in the absence or presence of LY294002 (10 mM). After 6 h of treatment, MG53 levels were assessed by western blot. *P < 0.05 vs. Control. &P < 0.05 vs. Insulin; n = 3.

**Figure 7:**
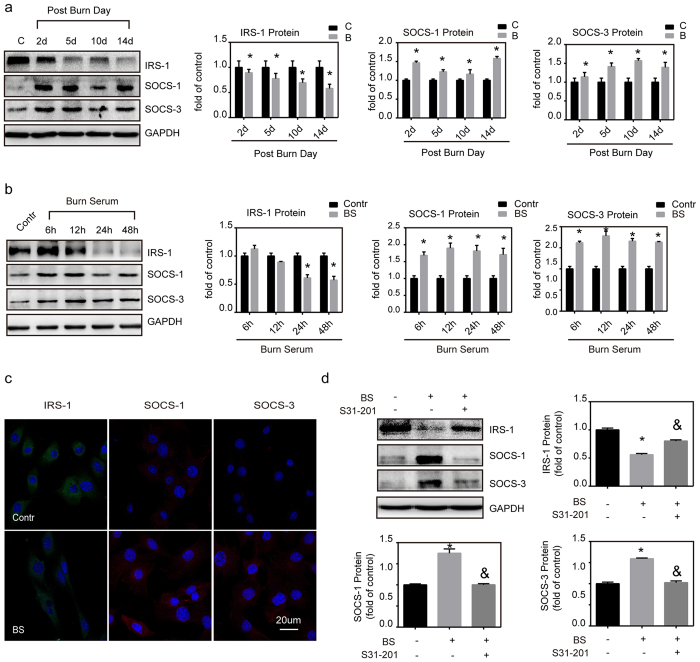
The roles of SOCS-1 and SOCS-3 in insulin insensitivity in skeletal muscle after burn. (**a**) Gastrocnemius muscles were collected at 2, 5, 10, 14 d after injury. Protein abundance of IRS-1, SOCS-1 and SOCS-3 were assessed by western blot using their antibodies. *P < 0.05 vs. Control; n = 3. (**b**) C2C12 myotubules were cultured for 48 h and then cultured with 2% burn serum. Protein levels of IRS-1, SOCS-1 and SOCS-3 were determined by western blot at 6, 12, 24, 48 h after burn serum treatment. *P < 0.05 vs. Control, n = 3. (**c**) C2C12 myotubules were cultured for 48 h and cultured with 2% burn serum. After 24 h of treatment, immunofluorescence microscopy was used to visualize the localization of IRS-1 (green), SOCS-1 (red) and SOCS-3 (red) (Scale bar, 20 μm). (d) C2C12 myotubules were cultured for 24 h and cultured with 2% burn serum in the absence or presence of SI3-201 (150 mM). After 24 h of treatment, the expression level of IRS-1, SOCS-1 and SOCS-3 protein were determined by western blotting. *P < 0.05 vs. Control. &P < 0.05 vs. Burn Serum; n = 3.

As illustrated in Fig. 6a,b, insulin could upregulate MG53 mRNA and protein levels in C2C12 myotubules when compared with controls, and that these effects could be abolished by inhibition of PI3K via LY294002. These results validate the notion that MG53 expression is regulated by IRS-1/PI3K signal pathway^17^. In this study, burned mice showed that IRS-1 expression in muscles began to decrease at 2 d after burn injury and that this decline remained up to 14 d after the injury, when compared with sham controls (Fig. 7a). The *in vitro* studies also showed that burn serum induced a significant decline in IRS-1 protein levels in myotubules when compared with the controls (Fig. 7b,c).

### Insulin insensitivity induced by Suppressor of cytokine signaling (SOCS)

It has been established that SOCS-1 and SOCS-3 are transcriptionally regulated by JAK/STAT signal pathway, and have been shown to block IRS-1/PI3K/Akt signal pathway. To investigate the roles of SOCS-1 and SOCS-3 in insulin insensitivity after burn injury, we demonstrated that both SOCS-1 and SOCS-3 were significantly increased in gastrocnemius muscles two weeks after burn injury (Fig. 7a). The *in vitro* studies also illustrated that burn serum increased the expression of both SOCS-1 and SOCS-3 proteins when compared with control myotubules cultured with sham serum (Fig. 7b,c). Moreover, we demonstrated that the burn serum-induced increase in SOCS-1 and SOCS-3 protein levels both returned to control values by inhibition of STAT with S3I-201, while IRS-1 began to increase significantly (Fig. 7d).

## Discussion

Musculoskeletal derangements, a well-known medical complication of burn patients, include muscle atrophy, muscle weakness and bioenergetic disorders, markedly contributing to the incidence of mortality and disability in burn patients^1^,^25^. This acquired systemic skeletal muscle myopathy, generally speaking, has been viewed as a result of muscles proteolysis causing by systemic inflammatory response syndrome (SIRS)^5^. However, the proteolytic pathways in skeletal muscles display persistent activation after burn injury, even when inflammatory factors in plasma are significantly decreased. Recently, abundant evidence indicates that sarcolemmal membrane injury is a major factor in the development of many myopathies^11^,^26^,^27^. However, the roles of sarcolemmal membrane damage in myopathy in burns remain unknown. A striking observation of our study was that burn injury induced a serious failure of plasma membrane repair, resulting in skeletal muscle membrane injury.

In this study, myofibers isolated from burn-injured mice displayed a significant increase in EBD staining via morphological and quantitative analyses, indicating that burn injury exacerbated skeletal muscle membrane injury. How could severe burns result in myofiber membranes damage? Membrane repair response is such a crucial process to repair the membrane of the injured myotubes in a timely and accurate manner that membrane damages are transient and rarely cause fatal injuries. *In ex-vivo*, we found that skeletal muscle myofibers from burn-injured mice displayed a disability to repair membrane disruptions following laser damage. *In vivo*, we used EBD to show that repair of damages, induced by treadmill exercise, is impaired in burn-injured mice, indicating that the membrane repair defect is not one unique to a UV laser injury, but one that arises under physiological conditions as well. Furthermore, compared with sham controls, the serum LDH and CK-MB levels in burn injury significantly increased after intramuscular injection of CTX, which also provided an effective marker of the skeletal muscle membrane repair defects in burn injury. Therefore, these findings *in vivo* and *ex-vivo* models suggest burn injury led to a significant failure of plasma membrane repair in skeletal muscle, which could be a major pathogenesis of burn-induced musculoskeletal membrane injury.

It is convinced that continuous release of intracellular “danger” signals or myokines from myocytes with defective membrane repair induces localized inflammation and exerts autocrine, paracrine and endocrine effects within the muscle itself as well as other organs to aggravate muscular bioenergetic dysfunctions, then resulting in muscle atrophy, muscle weakness, thus leading to myopathy^10^,^28^. Indeed, multiple forms of muscular diseases are caused by sarcolemmal membrane repair failure, such as DMD and diabetic myopathy. Interestingly, severe burns also induce a significant reduction of muscle mass, capacity, and bioenergetic dysfunctions, and these muscular changes in burn patients are similar to that showed in DMD and diabetic myopathy^11^,^27^, indicating that membrane repair failure underlie the burn-induced skeletal muscle membrane damage, and ultimately result in myopathy.

How could burn injury result in membrane repair failure? Numerous proteins have been demonstrated to participate in sarcolemmal membrane repair response, one of which is MG53. There are abundant studies showing that MG53 plays a major role in sensing the influx of extracellular oxidants and facilitating “patch vesicles” translocation of membrane repair machinery to initiate this repair response^12^,^15^,^16^,^29^,^30^. It has been reported that the mice lacking functional MG53 result in the development of muscular atrophy^15^,^19^. In particular, protein disulfide bond formation mediated by Cys242 is essential for MG53-mediated translocation of “patch vesicles” toward the disruption sites, as expression of cysteine mutation, C242A, results in nearly complete loss of MG53 dimerization and a significant failure of membrane repair^31^. Strikingly, MG53 dimer levels in skeletal muscles began to decrease at 5 d after burn injury, while MG53 expression showed no significant changes in these muscles at this time point. These results indicate that burn injury induces a perturbation in MG53 dimerization, which can be a key molecular mechanism leading to burn-induced musculoskeletal membrane repair failure.

To our knowledge, PDI has been demonstrated to participate in disulfide bonds formation between cysteine residues, and is essential for the dimerization of many proteins^32^. In this study, our *in vitro* findings showed MG53 dimer levels in myotubules were reduced by inhibition of PDI with 16F16 and thiomuscimol, indicating that PDI was an essential enzyme participating in the formation of disulfide bonds between MG53. Furthermore, we showed that the PDI activity in skeletal muscle was significantly decreased after burn injury. Therefore, these findings together indicate that burn-induced PDI inactivation, can inhibit MG53 dimerization, and finally, result in membrane repair failure. We further demonstrated that the NO levels in skeletal muscle significantly increased, and PDI was mediated by NO to produce a “SNO-PDI”. This NO mediated S-nitrosylation of PDI could be a possible cause of PDI inactivation in burn injury^22^,^23^. In addition, PDI can become oxidized and lose its ability to function as a disulphide bond-rearranging enzyme in the presence of ERS^24^. We demonstrated that CHOP, a major ERS marker, was significantly increased in skeletal muscles after burn injury, indicating that ERS also participated in burn-induced PDI inactivation.

In addition, we showed that the total MG53 content in skeletal muscle was reduced at the beginning of 10 d after burn injury. As a result, MG53-deficiency could be another molecular mechanism causing burn-induced sarcolemmal membrane repair defect^15^. In C2C12 myotubules, MG53 mRNA and protein levels could be elevated by insulin and abolished by PI3K inhibition using LY294002, indicating that burn-induced MG53-deficiency could be caused by insulin insensitivity. Indeed, skeletal muscle insulin insensitivity is a common phenomenon observed for prolonged periods of time after burn injury^18^,^33^,^34^,^35^. Thus, we believe that this burn-induced chronic insulin insensitivity participates in sarcolemmal membrane repair deficiency via MG53.

It has been established that IRS-1 is a major protein to transport the signal from insulin and its deficiency has been observed to be a common primary cause of persistent insulin insensitivity^18^. In this study, we illustrated that burn injury induced a persistent decline in IRS-1 protein levels in skeletal muscles, which validated the notion that IRS-1 proteins are a main target for the development of chronic insulin insensitivity in burn injury. Meanwhile, we demonstrated that both SOCS-1 and SOCS-3 were significantly increased in skeletal muscles two weeks after burn injury. Further, in our *in vitro* model system, we observed that burn serum rapidly enhanced SOCS-1 and SOCS-3 expression, and subsequently decreased IRS-1 protein. However, these effects were shown to be abolished by inhibition of STAT with S3I-201. These findings *in vivo* and *in vitro* together suggest that a high-grade of inflammation is induced after burn injury and that SOCS-1 and SOCS-3 mediated ubiquitin pathways could be involved in degradation of IRS-1 after burn injury, which finally results in prolonged insulin insensitivity.

The fact that membrane repair failure is a major pathogenetic mechanism of burn-induced myopathy cannot be undermined. In our opinion, promotion of endogenous MG53 and MG53-dimerization is a novel measure to repair sarcolemmal membrane and prevent burn-induced myopathy. In addition, Weisleder and colleagues recently demonstrated that the mice treated with recombinant human MG53 (rhMG53) displayed decreased muscle membrane damage and reduced muscle pathology without toxicity^19^. These results suggest that treatment with recombinant human MG53 could improve muscle membrane damage in burn injuries.

In summary, we provide evidence from *in vivo* and *ex-vivo* assays that show that the burn injury induces a significant failure of musculoskeletal membrane repair, which leads to skeletal muscle membrane damage. It is conceivable that this membrane repair failure could negatively affect musculoskeletal functions in burn patients. Furthermore, our results have identified, for the first time that the disorders in MG53 expression and MG53 dimerization are involved in musculoskeletal membrane repair defects as a result of burn injury. However, the pathogenesis of myopathy in burn patients is thought to be complex and multifactorial, and therefore, trials on human subjects are warranted to confirm the findings of our study, and their application to humans.

## Materials and Methods

### Reagents

Antibodies to MG53, IRS-1, SOCS-1 and SOCS-3 were obtained from Abcam (Cambridge, MA, USA). Antibodies to PDI and CHOP were obtained from Cell Signal Technology (Danvers, MA, USA). Antibodies to Glyceraldehyde 3-phosphate dehydrogenase (GAPDH) were purchased from KangChen Shanghai (Shanghai, China). FM1-43 dye was obtained from Biotium (Hayward, CA). Biotin–HPDP was obtained from Thermo Fisher Scientific (Waltham, MA USA), Pentobarbital, Buprenorphine and Lactated ringer’s solutions were obtained from Chongqing pharmaceutical industry (Chongqing, China). LY294002 and S3I-201 were obtained from Beyotime Biotechnology (Shanghai, China). Tyrode solution (5 mM KCl, 140 mM NaCl, 2 mM MgCl2, 2.5 mM CaCl2 and 10 mM HEPES (pH 7.2)) were obtained from Shanghai Biotechnology (Shanghai, China). Unless marked, all reagents were obtained from Sigma-Aldrich (St,Louis, MO, USA).

### Animal models

One hundred eight male BALB/C mice (6 weeks old, 25 g to 30 g body weight) were obtained from the Third Military Medical University Laboratory Animal Centre. The mice were raised in individual cages under 22–25 °C and with a 12 h light-dark cycle. The mice were used to the environment with a standard diet for a week prior to the experiment. One hundred eight mice were randomly divided into two groups, control (C) and burn-injury (B) groups. The mice from B group were subjected to 30% total body surface area (TBSA) of full thickness burn as previously described^36^. Briefly, mice were anesthetized with 1% pentobarbital (40 mg/kg of body weight), treated with buprenorphine (1 mg/kg body of weight) for analgesia and their backs were shaven with hair clippers. Mice were placed in a model designed to expose 30% of their dorsa skin to a hot water bath at 90 °C for 10 s. After injury, mice were intraperitoneally injected with lactated ringer’s solution (50 ml/kg of body weight). Sham injured mice were anesthetized, shaven, and intraperitoneally injected with lactated ringer’s solution. Each treatment group (sham control, 2, 5, 10, and 14 d after burn) employed twelve animals. All animal experimental protocols were approved by the Third Military Medical University Animal Care Committee according to the National Institutes of Health Guide for the Care and Use of Laboratory Animals (NIH publication number 8023, revised 1978).

### Cell culture

Our C2C12 were obtained from ATCC (Manassas, VA, USA) and cultured by Dulbecco’s modified Eagle’s medium (DMEM) supplement with 10% fetal bovine serum (Gibco) and 1% penicillin/streptomycin in a 5% CO_2_ incubator at 37 °C. Sequentially, confluent C2C12 myoblasts were differentiated for 48 h by incubation with DMEM supplemented with 2% horse serum. (a). Experiment is divided into control (Contr) and 16F16 (50 μM) treated groups. (b). Experiment is divided into control (Contr) and thiomuscimol (25 μM) treated groups. (c). Experiment is divided into control (Contr), insulin treated (INS) and insulin+PI3K inhibitor treated (INS+LY294002) groups. (d). Experiment is divided into control (Contr), burn serum treated (BS), burn serum+STAT inhibitor treated (BS+S3I-201) groups.

### Membrane repair assay *in vivo*

(1) EBD (1%, 10 ml/kg of body weight, Sigma-Aldrich) was intraperitoneally and intramuscularly injected into burn-injured and sham control mice 12 h prior to exercise training. Mice were subsequently subjected to treadmill exercise (Zhenghua Instruments, AnHui, China) at 15 m/min for 90 min. Then, the mice were sacrificed by cervical dislocation and gastrocnemius muscles were intactly excised. Gastrocnemius muscles were sectioned and imaged on a Zeiss-LSM 510 confocal microscope. Furthermore, gastrocnemius muscles were soaked in formamide for 48 h at 55 °C in dark place. We next used the spectronic 610 spectrophotometer (Milton Roy) to measure the optical density of EBD in muscle. (2) We further intramuscularly injected CTX (10 uM, 10 ml/kg of body weight, Sigma-Aldrich) into burn-injured and sham control mice. At 12 hours post-injection, mice serum was collected for ELISA tests for the levels of LDH and CK-MB (Sigma-Aldrich)^15^,^19^,^29^.

### Membrane repair assay *in ex-vivo*

FDB muscles were surgically intactly excised in a Tyrode solution. FDB muscles were digested in Tyrode solution supplemented with type I collagenase (2 mg/ml), for 60 min at 37 °C with gentle rocking. After digestion, FDB muscles were further dissociated to single fibers by passing them through pipettes with small diameter. Single myofibers were plated onto plastic-bottomed dishes (Bioptechs) in Tyrode solution. Then, a 5 × 5 pixel area of the plasma membrane was irradiated with UV-laser (Enterprise, 80 mW, 351/364 nm) for 5 s in Tyrode solution supplemented with 2.5 μM FM1-43 dye. By using a Zeiss-LSM 510 confocal microscope, a 200 μm^2^ area adjacent to the damage site was utilized for measuring FM1-43 dye influx fluorescent intensity. Data is presented as ΔF/F_0_^20^.

### RT-PCR

Trizol (Invitrogen, CA, USA) was used to isolate total RNA in C2C12 myotubules. RNA was used as a template for cDNA synthesis using Moloney Murine Leukemia Virus reverse transcriptase (Invitrogen, CA, USA). RT-PCR was executed using Taq polymerase and the following primers (Invitrogen, CA, USA): MG53 (forward, reverse) primers include: 5′-ACTGAGCATCTACTGCGAGC-3′ and 5′-ACGATGACCACGGTGAGAAC-3′; GAPDH (forward, reverse) primers include: 5′-CTGCGACCACCAACTGCTTAGC-3′ and 5′-CTTCACCACCTTCTTGATGTC-3′.

### Immunohistochemistry

Gastrocnemius muscles were sectioned (10 μm thick) using a cryostat (CM1900, Leica, Wetzlar, Germany) at −20 °C. The sections were incubated with 2% H_2_O_2_ in absolute methanol for 30 min to eliminate endogenous peroxidase activity, and then blocked with 1% bovine serum albumin (BSA) phosphate-buffered saline (PSA) containing 0.1% Triton X-100 for 60 min. The muscle sections were incubated at 4 °C for 12 h with antibodies to MG53 (1:200), followed by incubation with secondary antibodies. Diaminobenzidine (DAB) was detected and imaged by Axiovert 200 microscope (Zeiss).

### Western blot analysis

The extracted proteins were separated using sodium dodecyl sulphate-polyacrylamide gel electrophoresis (SDS-PAGE), and transferred to a polyvinylidene fluoride (PVDF) membrane. These membranes were incubated at 4 °C overnight with anti-MG53 (1:200), anti-PDI (1:500), anti-CHOP (1:200), anti-IRS1 (1:1000), anti-SOCS-1 (1:200), anti-SOCS-3 (1:1000) and GAPDH (1:5000) antibodies, followed by washes with Tris-Buffered Saline Tween (TBST) and incubation with appropriate secondary antibodies. Following washes with TBST, protein bands were detected using enhanced chemoluminescence ECL (Healthcare, Buckinghamshire, United Kingdom).

### Immunofluorescent microscopy

C2C12 myotubes were cultured on coverslips, and then fixed in 3.7% paraformaldehyde solution for 10 min, permeabilised with 0.1% Triton X-100 in PBS for 30 min, and blocked in 2% BSA in PBS for 30 min. C2C12 myotubes monolayers were incubated with antibodies to SOCS-1 (1:1000), SOCS-3 (1:1000), IRS-1 (1:1000) for overnight at 4 °C, followed by incubation with secondary antibodies for 1 h at 37 °C. The cell nuclei were stained by DAPI (blue) at a concentration of 1.43 μM.

### Biotin-switch assay

SNO-PDI was tested using the biotin-switch assay. Briefly, protein samples were added to a mix with 20 mM methylmethanethiosulfonate and 2.5% SDS in HEN buffer and were incubated at 50  °C for 1 h. Acetone were used to remove methylmethanethiosulfonate, then we used 1 mM ascorbate to reduce free thiols. The formed thiols were linked to the biotin–HPDP. The biotinylated proteins were pulled down, and SNO-PDI remaining in the precipitation was detected by western blotting.

### PDI activity assay

The PDI activity was detected as described previously. Briefly C2C12 myotubes lysates were mixed with buffer (1 mM DTT, 30 μM insulin, and 3.0 mM sodium EDTA, in a buffer containing 100 mM sodium phosphate). Precipitation of insulin B chain was measured by an increase in absorbance at 650 nm.

### Statistical analysis

All data were presented as mean ± standard error of the mean (SEM). Statistical analysis for these experimental data were evaluated by significance using one-way analysis of variance (ANOVA) and corrected for multiple comparisons. Results with P values of <0.05 were considered to be significant. Statistical analysis was performed with SPSS software (version 13.0).

## Additional Information

**How to cite this article**: Wang, C. *et al*. A novel perspective for burn-induced myopathy: Membrane repair defect. *Sci. Rep*. **6**, 31409; doi: 10.1038/srep31409 (2016).

## Figures and Tables

**Table 1:** Quantitative analysis of membrane integrity and repair capacity (mean ± SEM; *P < 0.05 vs. Control).

Content	EBD (non-exercised, ng/g)	EBD (exercised, ng/g)
Control	20.07 ± 0.93	23.47 ± 2.46
Burn injury	27.93 ± 2.94*	42.48 ± 2.40*

EBD was intraperitoneally injected into burned (Post Burn Day 14) and control mice (n = 5 mice per group). Quantitative assessment of total absorbance of Evan’s Blue Dye (EBD) extracted from gastrocnemius muscle bundles of mice subjected to treadmill exercise or not.
